# Ecological Networks in Urban Forest Fragments Reveal Species Associations between Native and Invasive Plant Communities

**DOI:** 10.3390/plants11040541

**Published:** 2022-02-17

**Authors:** Sonali Chauhan, Gitanjali Yadav, Suresh Babu

**Affiliations:** 1 School of Human Ecology, Ambedkar University Delhi, Delhi 110006, India; sonalichauhan@aud.ac.in; 2 Centre for Urban Ecology and Sustainability (CUES), Ambedkar University Delhi, Delhi 110006, India; 3 National Institute of Plant Genome Research, New Delhi 110067, India

**Keywords:** novel ecosystems, complex networks, tree communities, *Lantana camara*, *Prosopis juliflora*

## Abstract

Forest fragments are characteristic features of many megacities that have survived the urbanisation process and are often represented by unique assemblages of flora and fauna. Such woodlands are representations of nature in the city—often dominated by non-native and invasive species that coexist with resilient native congeners and purposefully introduced flora. These forest fragments also provide significant ecosystem services to urban society and therefore, understanding their compositional patterns is of considerable importance for conservation and management. In this work, we use a complex network approach to investigate species assemblages across six distinct urban forest fragments in the South Delhi Ridge area of the National Capital Territory, India. We generate bipartite ecological networks using conventional vegetation sampling datasets, followed by network partitioning to identify multiple cliques across the six forest fragments. Our results show that urban woodlands primarily form invasive–native associations, and that major invasive species, such as *Prosopis juliflora* and *Lantana camara* exclude each other while forming cliques. Our findings have implications for the conservation of these urban forests and highlight the importance of using network approaches in vegetation analysis.

## 1. Introduction

The city of Delhi (National Capital Territory) in North India is constructed over 1100 square kilometres of erstwhile Dry Thorn Scrub Aravalli vegetation, which comprises agricultural lands and the wetlands of the Yamuna river [1]. Over the past 500 years, Delhi has been built and rebuilt several times with major changes in the landscape during the Mughal and British periods. Among these changes were general beautification efforts and the creation of parks. The Delhi Ridge Forest consists of sections known as the Northern, Central, South-Central and the Southern Delhi Ridge, all of which are fragments of the erstwhile Aravalli vegetation that survived these transformations. The ‘Delhi Ridge’ (as these forest fragments are referred to) thus includes remnants of the Aravalli woodlands that have survived urbanisation. The contemporary literature on ecology refers to such urban ecosystems as ‘novel ecosystems’ [2], in that they represent unique formations that are not fully understood by ecosystem ecologists and pose challenges to the general principles of community ecology. The Delhi Ridge Forest is known to have been overrun by invasive species such as *Prosopis juliflora* and *Lantana camara*, and much of the vegetation now consists of a combination of invasive species, exotics or agricultural escapes, apart from native Aravalli species.

*Prosopis juliflora* was first introduced in Delhi around the 1900s by William R. Mustoe, an expert gardener during the colonial afforestation program of Delhi [3,4]. Even post-independence, it remained an important part of greening efforts [5]. Owing to its drought tolerance and rapid growth, this species was widely introduced in several Indian states including Gujarat, Madhya Pradesh, Rajasthan and Delhi. Later on, it invaded agricultural fields, pasturelands and forest areas [6,7]. Today it is amongst the most aggressive plant invaders and is considered a nuisance in several arid and semi-arid areas of Asia, Africa and North America. It has been reported that *Prosopis juliflora* changes the physicochemical and nutrient profiles of soils, and has allelopathic characteristics that lead to suppression of understory vegetation [8,9,10,11]. In Delhi Ridge, *Prosopis juliflora* has been cited as the main reason for the disappearance of native tree species such as *Prosopis cineraria* [11].

The history of the arrival and ecological impacts of *Lantana camara* in Delhi have not been well studied, despite this species being amongst the top 100 most invasive plants in the world [12]. It is estimated to have been introduced in the 1800s as an ornamental plant in India due to its showy flowers [13]. It is a fast-growing shrub, produces many seeds and can also propagate vegetatively. *Lantana camara* forms dense thickets that crowd out seedlings of native species, has a wide environmental tolerance range and is reported to have the allelopathic potential [14,15]. It has been reported as the most invasive weed in national parks and nature reserves of India, wherein it colonises forest gaps, periphery and other disturbed areas [14]. Urban forests and woodlands tend to be much smaller in size and have a high degree of internal fragmentation compared to the more protected reserves of non-urban regions. Accordingly, urban forests become ideal hotspots for the colonisation and spread of invasive species such as *Lantana camara*. Species distribution maps of both *Lantana camara* and *Prosopis juliflora*, with data presented on a temporal scale are publicly available in the GBIF (the Global Biodiversity Information Facility) database and interested readers can find interactive temporal distribution maps at these URLs [16,17].

Urban forests are particularly prone to invasion due to fragmentation, changes in environmental conditions, heavy pollutant load and the constant inflow of non-native species [18,19,20]. There is increasing evidence that urban nature plays a significant role in modulating the microclimate, and enhancing the quality of life in cities [21,22,23]. The actual relationship between these species however, is less known, and several questions remain unanswered. For instance, do invasive species decimate native vegetation? If so, do the present forest patches consist of singular stands of invasives? How do native species respond to the propagule pressure of invasive species? In short, this work was undertaken with the aim of understanding what kind of species associations form as a result of the combined impact of urbanisation, biological invasions and active use by local communities.

In the context of network theory, a complex network is a graph (network) with non-trivial topological features that are often associated with robust or real-world systems such as computer networks, disease networks, technological networks, climate networks and social networks [24].The use of network approaches in ecological studies has increased over the past decade but these are largely focused on mutualistic networks (plant–pollinator) of species or the stability of ecological networks in which species’ interactions are already known [25,26,27,28].The novelty of this work lies in making use of a network approach to supplement traditional multivariate analyses in plant ecology for the visualisation of special assemblages. Since the early 20th century, the identification of plant communities and the patterns of their organisation have been some of the core research areas in vegetation ecology [29,30]. Ordination methods are commonly used for the identification of communities and vegetation–environment correlates using techniques such as cluster analysis, principal component analysis (PCA), non-metric multidimensional scaling (NMDS), correspondence analysis (CA), etc. These techniques are sensitive to species abundances, some of which are biased towards rare species, or species-rich areas while others are based on the assumption of linearity or appropriate for unimodal distribution. Each of these techniques has advantages and disadvantages and requires careful consideration of the various algorithms used based on the type of data; compute-intensive nature and software availability. Biplots produced by ordination techniques may be efficient in visualising community structure, but these are cumbersome in projecting large species associations and individual interactions [31,32,33].

In this work, we propose a novel approach of adding graph theoretical methods to detect and visualise ecological communities, and we use this approach to explore species associations across six distinct urban forest fragments of the South Delhi Ridge. We then use network partitioning methods to identify and compare topologically significant ‘cliques’ formed by native and/or invasive species in these forests. Our results provide new insights into competitive exclusion apart from identifying emergent invasive species that are likely to become established in these urban ecosystems. Taken together, our work offers a new and parallel method of simultaneously investigating patterns at the level of both community and individual species.

## 2. Results

### 2.1. Study Area Networks

**Figure 1:**
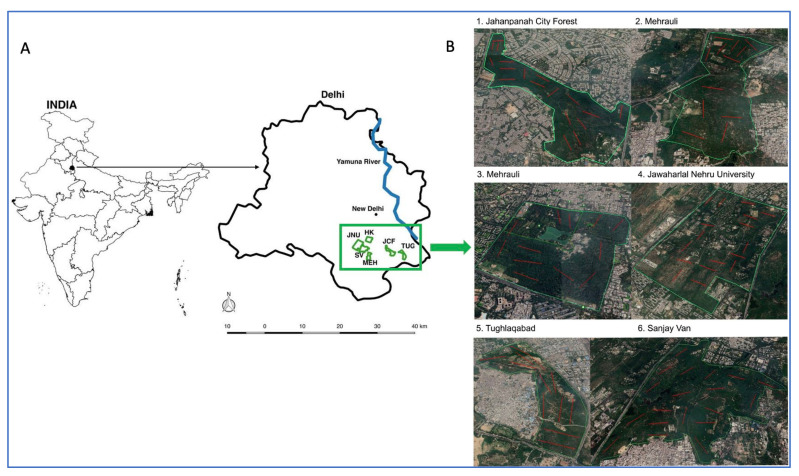
Study area map indicating the six urban forest fragments investigated in this work, in terms of (**A**) location and (**B**) landscape topology. Red lines in (**B**) indicate line transects placed in each sampling site using Google Earth Pro.

We identified over 5500 associations among 57 native, introduced and invasive plant species spread across the six study sites in the urban forests of the South Delhi ridge, New Delhi. These six sites have been identified as described in the Methods section and are depicted on the Delhi NCT map in Figure 1. The plants were categorised into 50 native, four invasive and three introduced (non-native) species, all of which were named and assigned short codes as listed in the Table S1. The four invasive species, along with their codes, are *Lantana camara* (LC), *Prosopis juliflora* (PJ), *Opuntia* (OP), and *Leucaena leucocephala* (LL). The three non-native introduced species include *Morus alba* (MOA; Mulberry), *Cassia tora* (CT) and *Azadirachta indica* (AI or Neem).

**Figure 2:**
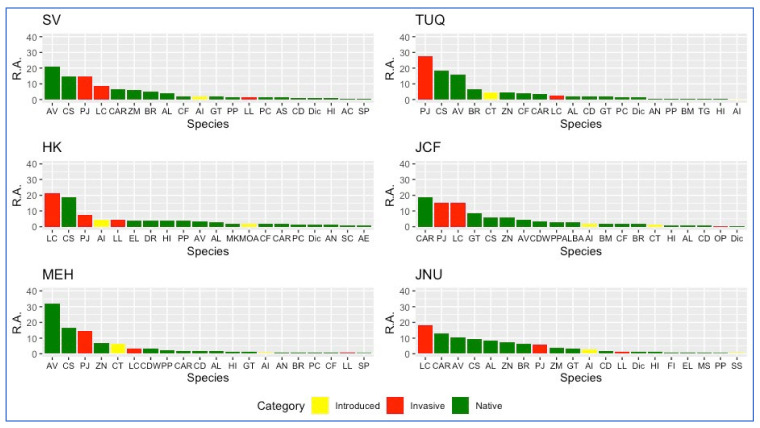
Ranked abundance of native (green), invasive (red) and yellow (introduced) species in each study site. For species codes, see Table S1.

**Figure 3:**
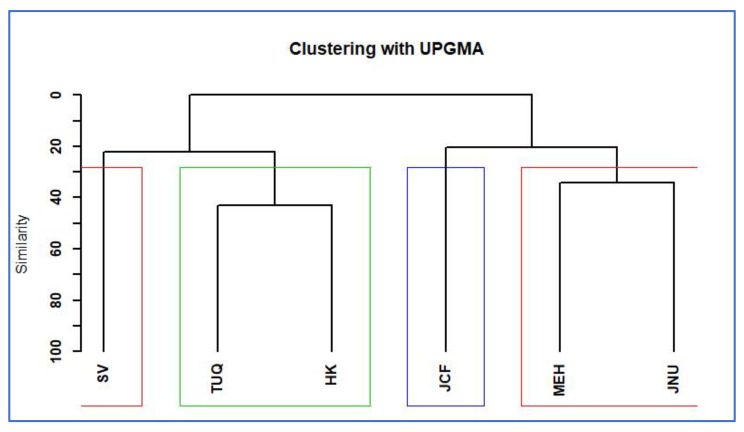
Cluster analysis between sampling sites using Bray Curtis dissimilarity index.

Each study site represents a distinct ecological community as established by a rarefaction test, and relative abundance was measured across these communities. The six graphs in Figure 2 depict ranked abundance patterns, indicating the extent to which invasive species appear to dominate each sampling site, as compared to native or introduced species. For example, one or more of the top three species in each community are invariably invasive, with three study sites (TUQ, HK and JNU) having invasive *Lantana camara* or *Prosopis juliflora* as the most abundant species in the community. In case of SV, HK and JCF, both invasive species (LC and PJ) are among the most abundant. The three introduced species are generally low in abundance, although at least one of these appears among the top five most abundant species in the TUQ, HK and MEH communities. A UPGMA-based cluster analysis of the communities across the six sites revealed these to be roughly divisible into two groups, namely, SV-(TUQ-HK) and JCF-(JNU-MEH). These relationships are depicted as a dendrogram in Figure 3 using the Bray Curtis dissimilarity index as described in Methods. The dendrogram in Figure 3 reveals greater compositional overlap between TUQ and HK as compared to JNU and MEH, although their most abundant invasive species are distinct (TUQ forest fragment is predominated by *Prosopis juliflora* while the HK forest fragment has *Lantana camara* as the major invasive). The two remaining communities, namely, JCF and SV, are distinct in the dendrogram, but these also stand out among the other four communities, in terms of having high abundance of both *Prosopis juliflora* and *Lantana camara* as can be seen in Figure 2.

**Figure 4:**
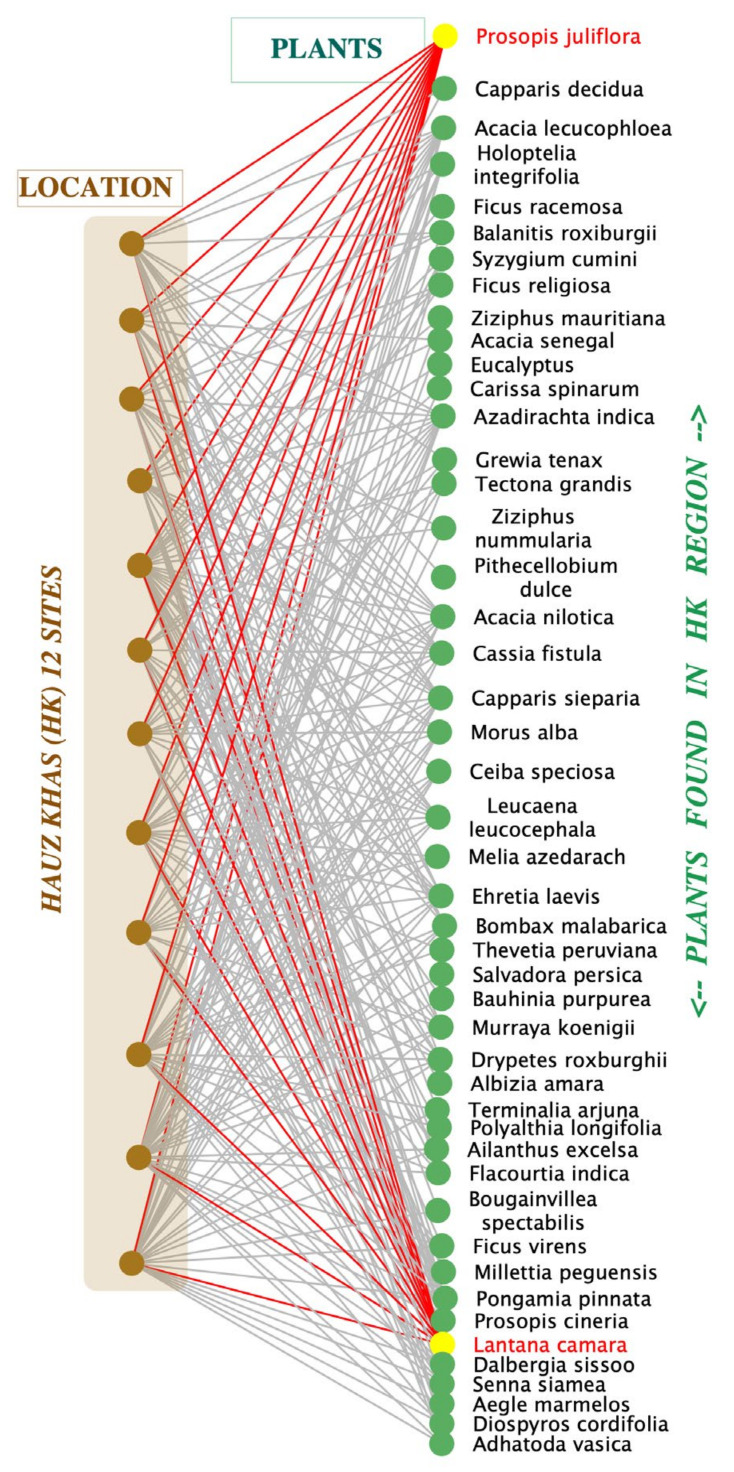
Bipartite species association network of Hauz Khas (HK) Forest fragment. Circles (nodes) represent plants (green) or location/transects (brown), and lines (edges) connect plants found at a location. This network has 256 associations (edges) between 49 plant species located across 12 transects of 200 m each, in the HK Ridge Forest. Note how the invasive species (yellow highlighted nodes) *Lantana camara* and *Prosopis juliflora* are present in all transects (red edges) of this forest fragment.

In order to understand the six forest fragments in terms of their native and invasive plant communities, we generated six bipartite species association networks, one for each forest fragment, as described in Methods. Nodes in these networks represent one of two independent sets or ‘partites’, namely, plants and transects, in order to retain both location and species identity. Accordingly, any two species found on a given transect were considered ‘associated’ in the bipartite networks, as shown in Figure 4, the study area network of Hauz Khas ridge forest (HK). As can be seen in this network, the HK forest fragment has 256 edges or associations between 49 species identified across 12 transects. Each edge in these bipartite (two-mode) networks ties a plant (green) to the location (brown) where it was found. Each node is either a transect labelled by the forest code (i.e., HK1 to HK12 in case of HK) or a woodland species that has been assigned a short code (two to four letters) based on its species and genus names (Table S1).

In general, invasive species were found to be present extensively in each forest fragment, as evidenced by their occurrence across transects. For instance, note how the invasive shrub *Lantana camara* (highlighted in yellow) is present in each of the 12 HK transects (red edges) in Figure 4. Similarly, *Prosopis juliflora* was also present in all transects investigated. In contrast, other native species such as *Acacia nilotica* (AN) or naturalised species like *Cassia tora* (CT) are often present in low numbers as evident from the relative abundance of these species across the study sites (Figure 2). This pattern holds true in each of the six forest fragments investigated and supports the theory of local decline and displacement of native species in urban forest fragments. However, the coexistence of native and invasive species needs to be investigated further to understand their associations and to explore patterns across different sites. The next section addresses this aspect to understand the nature and extent of native species displacement across the forest fragments investigated.

### 2.2. Distinct Native–Invasive Communities

**Figure 5:**
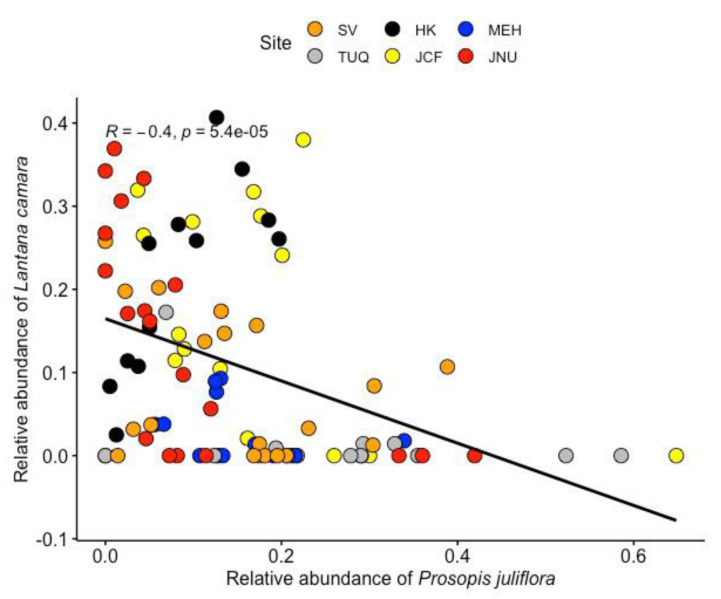
Correlation between the relative abundance of *Prosopis juliflora* and *Lantana camara* reveal a significant negative relationship.

Community detection was performed as described in Methods and we found about 25 species-specific community cliques across the six urban forest sites. Three to six distinct cliques were found in each forest fragment based on the Glay partitioning algorithm, as can be seen in Table 1. Each ‘clique’ is identified as a set of densely connected nodes in the network, which in turn represents woodland species comprising various combinations of native, introduced and invasive plants. Interestingly, all forest fragments in the South Delhi ridge have cliques with dominant invasive members, with two sampling sites (SV and HK) having each of the three invasive species forming their own cliques. Some native trees form communities without any invasive species, but such ‘native–native’ communities are few, and they tend to have fewer species than the native–invasive cliques. Another significant pattern among these species associations, in terms of invasives, is that the two most aggressive plant invaders of this region, namely, *Lantana camara* and *Prosopis juliflora* always occur in distinct communities, never together in the same clique. The correlation was tested as described in Methods and is depicted in Figure 5, revealing a significant negative association between *Lantana camara* and *Prosopis juliflora*, reaffirming that these two major invasive species of the region exclude each other, which supports the competitive exclusion theory.

**Figure 6:**
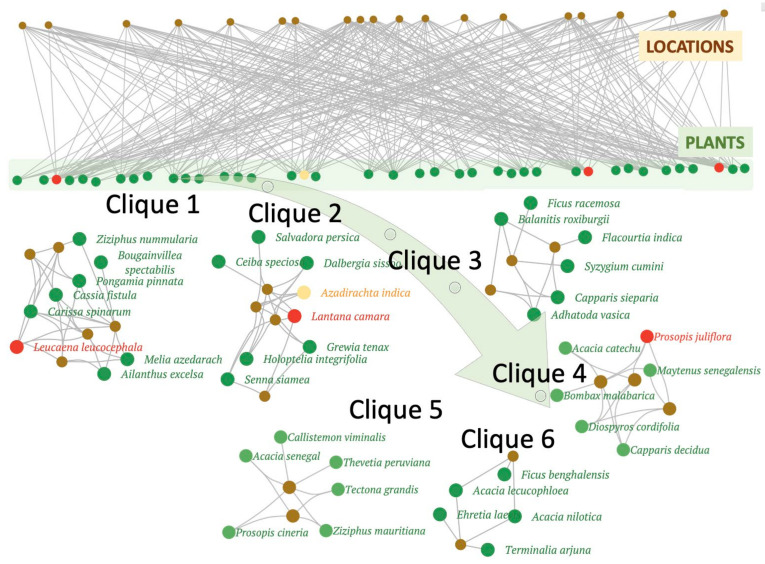
Community detection in the Sanjay Van (SV) Forest fragment. The upper panel depicts the complete bipartite species association network with 277 edges (grey lines) among 42 plant species (green circles) found across 19 transects (brown nodes). The lower panel depicts the six distinct cliques derived from the topology of this forest fragment, of which three are dominated by invasive (red) species *Leucaena leucocephala* (LL), *Lantana camara* (LC) and *Prosopis juliflora* (PJ).

As a case study, Figure 6 shows the six species association cliques identified in the SV forest fragment, and this is one of the communities with the largest number of cliques. Of the six cliques in the SV region, three (cliques 1, 2 and 4) have a dominant invasive member. The remaining three cliques are composed of native–native species associations but the number and size of such communities that have remained invasion-free are often small, and this may have a bearing on the stability and robustness of each of the six forest fragments. In order to be able to compare the six forest fragments, and to identify ecologically meaningful species associations, each of the six partitioned networks was standardised for community analysis. Before performing any community analysis, the Cytoscape ‘Glay’ plugin transforms the input network into a simplified model, with edge directionality, duplication and self-looping removed. This enables network standardisation, making the resultant community structures from different community detection algorithms comparable. This was followed by identification of keynote species or hub species by using the graph theoretical clustering algorithm, MCODE, based on vertex weighting by local neighbourhood density and outward traversal from a locally dense seed node to isolate the dense regions according to given parameters. The top-ranking cliques of this forest, along with their respective hub species are listed in Table 1, with invasives highlighted in bold font. Thus, dominant species for each clique identified as ‘hubs’ represent trees/shrubs that tend to co-occur more often than by random chance. A pattern can be observed among the 25 cliques listed in Table 1, (as well as other smaller cliques), indicating that the two major invasives tend to avoid each other and form exclusive groups with native species. The next section addresses the specificity, if any, of such invasive–native associations.

### 2.3. Patterns in Species Association

**Figure 7:**
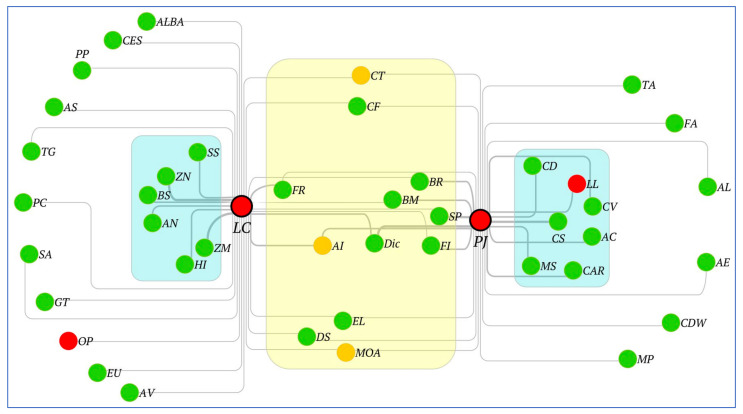
Distinct species associations of the two most aggressive invasives *Lantana camara* (LC) and *Prosopis juliflora* (PJ), both depicted as large red nodes with black borders. The two invasives have shared associations (yellow cluster) as well as distinct species associations with native (green), introduced (orange) species across the six investigated forest fragments. Edge weights represent the number of times a given association was observed.

Table 1 and Figure 6 in the previous section indicate how native species in urban forest fragments form distinct communities, some of which are dominated by ‘hubs’ that may comprise at most, one invasive species. From a management point of view, it would be interesting to understand the preference for native species, if any. The species association networks and the identified cliques of the six forest fragments enabled us to perform this analysis for each plant invader. Towards this, all 25 study area network cliques (listed in Table 1) were superimposed and the pairwise species associations of *Lantana camara* (LC) and *Prosopis juliflora* (PJ) were extracted from each community. This data was then visualised jointly in order to estimate the overlap between native species found to be associated with the two major invasives, as shown in Figure 7. Two main types of assemblages are discernible in this figure depicted in yellow and blue-shaded modules, respectively, of which the former represents the shared associations between LC and PJ, while the latter (blue) modules represent the assemblages unique to either LC or PJ. Furthermore, the nodes or species in the shared (yellow) module have lower edge weights as compared to the species in the non-shared or exclusive (blue) modules. Species that form common or shared associations include *Grewia tenax* (Gre), *Acacia leucophloea* (AL) *Acacia nilotica* (AN), as well as the three introduced species *Morus alba* (MOA; Mulberry), *Cassia tora* (CT) and *Azadirachta indica* (AI or Neem). However, it may be noted that these common woodland species are never found with ‘both’ LC and PJ, in the ‘same’ location/forest fragment.

Species in the blue modules of Figure 7 represent two exclusive sets of plants, preferred by *Lantana camara* and *Prosopis juliflora*, that are not shared with each other, such as for example *Carissa* (CAR) for PJ and *Tectona grandis* (Teak) for LC. Similarly, *Acacia leucophloea* is preferentially associated with *Prosopis juliflora* while *Acacia nilotica* forms species associations with *Lantana camara*. Interestingly, the invasive *Leucaena leucocephala (LL)* is present in the blue module of *Prosopis juliflora*, suggesting an invasive–invasive association that was not observed earlier in the individual study area networks. A comparison of the relative strengths of each of these two sets of associations, reveals that the blue (exclusive) modules are stronger than the shared (yellow) assemblages in terms of both module size and edge weights. This observation implies that shared associations may be transient or weak in nature, as compared to the exclusive associations formed by either *Lantana camara* or *Prosopis juliflora* in urban forests.

Looking closely at the preferential associations of these two invasive species (Figure 7) reveals some interesting insights. Firstly, the distinct sets of congeners for each invasive can further be divided into two subsets; one that is more preferred (blue module), as compared to the outer, more peripheral (white region) species with smaller edge weights, as these associations are detected less frequently. When compared with Table 1, the species in blue modules were often found among hubs in the 25 cliques. However, as noted earlier, there is not a single native species that is preferentially associated (thick edges) with both invasive species, providing further evidence for competitive exclusion, and this is discussed further in the next section. Another notable pattern from Figure 7 is that the invasive shrub *Lantana camara* tends to exclude other native shrub species and is only preferentially associated with one native shrub, i.e., *Ziziphus nummularia* (a berry). *Lantana camara* tends to form associations with small and medium-size tree species such as *Pongamia pinnata (PP)*, *Holoptelea integrifolia (HI)*, *Ziziphus mauritiana (ZM).* On the other hand, the invasive tree species *Prosopis juliflora* forms large groups with several native trees and shrubs and it is preferentially associated with non-native species (*Thevetia*). *Prosopis juliflora* is preferentially associated with several native trees such as *Ehretia laevis*, *Salvadora persica*, *Balanites roxburghii* and *Acacia leucophloea*. *Prosopis juliflora* forms associations with native shrubs *Capparis sepiaria*, *Adhatoda vasica*, *Carissa*. It may be noted that while *Prosopis juliflora* and *Lantana camara* exclude each other mutually, they are found to co-exist with other invasive species such as *Opuntia* (OP; cactus) or *Leucaena leucocephala* (LL), thus forming potential invasive–invasive cliques. However, OP and LL have not yet been identified as fully invasive in these habitats, and a closer look at Table 1 suggests that LL is likely to become a more aggressive invader in this region, as it was identified as the dominant invasive in at least two communities, namely, the SV and HK forest fragments. In two other cliques, it was identified as a non-hub member of PJ-associated cliques. On the other hand, OP was found in very few cliques, and may be considered as a borderline non-native species that is more likely to become naturalised in this region. In summary, the native species common to both invasives are either (a) preferentially associated with one invasive (blue boxes in Figure 7), or (b) equally rarely observed for both invasives (yellow box in Figure 7, but these are weaker edges).

## 3. Discussion

Urban flora is known to be largely composed of migrants or non-native species that become part of urban ecosystems as escapes from horticulture, forestry, agriculture sectors or enter through transport networks [34,35,36,37]. These species further form associations in different habitats types that range from being complete man-made (gardens, roadsides, parks, etc.) to semi-wild such as urban forests [38,39,40]. The urban forest fragments of South Delhi ridge are the key source of ecosystem services to the city but are constantly at risk of being converted to homogenised, species-poor habitats dominated by invasive species. This observation was supported in the present study in Section 2.1 (Figure 2 and Figure 3), where each sampling unit was found to be dominated by one or more invasive species, and overall, *Prosopis juliflora* and *Lantana camara* were identified as the most dominating invasive species in all six study sites. These observations reveal that the process of plant invasion is much more complex in urban forests, as there are multiple invasive species involved.

Further investigation and partitioning of study area networks in Section 2.2 (Table 1 and Figure 6) revealed that the two dominant invaders *Lantana camara* and *Prosopis juliflora* exclude each other and form distinct native–invasive, native–native and native–non-native species associations. This competitive exclusion between the two invaders could be a result of the difference in their habitat requirements. *Lantana camara* is a light-demanding understory shrub, that requires moisture and thick soil, while, *Prosopis juliflora* is a tree species known for its drought tolerance and ability to grow in rocky terrain, both species are also known to have allelopathic characteristics. These distinct high-fidelity native–invasive cliques suggest that plant invasion alters community composition by forming novel assemblages instead of wiping out the native flora, thereby acting at the community level by altering species associations. *Prosopis juliflora* is already known to have a differential impact on native species, altering community composition by forming associations with few ‘weedy’ native species [9,41,42]. *Prosopis juliflora* has a more detrimental effect on annual species than perennials and forms associations with species of ‘disturbed forests’, and has been reported to negatively affect ‘higher order seral’ species [41,42]. Our results also affirm this ecological alliance between native and invasive species. *Prosopis juliflora* is a nitrogen-fixing tree legume, capable of altering soil characteristics, thereby having a competitive advantage [43]. In addition, it is reported to have higher soil microbial biomass and mycorrhization intensity as compared to native tree species [44]. It alters community composition by forming associations with few native species and it has been suggested that in the Delhi Ridge, *Prosopis juliflora* is the main reason for the disappearance of native tree species [9,11]. On the other hand, *Lantana camara* forms a dense canopy that once established, not only limits native tree seedling recruitment but also inhibits the growth of other understory vegetation, forming small groups in comparison with *Prosopis juliflora*. *Lantana* is a gregarious shrub known for negatively affecting native species by outcompeting them for scarce resources and possessing allelopathic attributes. It forms impenetrable thickets, thus crowding out seedling recruitments. This light-loving plant often begins colonisation in forest edges and gaps, and takes advantage of disturbance events as it can quickly regenerate following fires as well as chopping or cutting [15]. Thus, in comparison to *Prosopis juliflora*, *Lantana camara* was observed to form smaller native–invasive cliques with almost no association with native shrubs. When an invasive species enters the ecosystem, it gradually takes over the assemblages and reduces the number of species in those clusters. The continuous inflow of non-native species and changing environmental conditions diverts historical assemblages to new associations, significantly influencing ecosystem structure and function [45,46,47]

Recent research on urban ecology often categorises these new associations as novel and views them as an opportunity to study ecosystem change. Thus, these ‘novel ecosystems’ usually describe an assemblage of non-native and native species that never existed before in an ecosystem. Novel ecosystems are composed of a non-historical species configuration that arises due to anthropogenic environmental change, land conversion, species invasion or a combination of the three. They are created as a consequence of human activity but do not depend on human intervention for their maintenance [46]. Under the framework, green spaces can be categorised into three categories, i.e., historical, hybrid and novel ecosystems. A hybrid ecosystem is similar to a historical system based on its function and species composition, while a novel ecosystem is defined as a system that has crossed a threshold beyond which ecological and social processes stops it from returning to its historical state. Novel ecosystem frameworks argue that the focus of ecologists should shift from patterns to processes and conservation practices should not focus on ‘fossilising nature’ but rather work towards restoring ecosystem function [46]. While it is too early to categorise the Delhi Ridge as a hybrid or novel ecosystem, such a framework can help provide more achievable targets in terms of species conservation in an urban context, keeping in mind the social, political and ecological conditions amidst which these forests are located. Many case studies have pointed out that some invasive species act as transformers to establish positive feedback loops by way of increased biomass, nitrogen fixation, etc. that may move an ecosystem to an alternate stable state wherein maintaining diversity becomes a near-impossible task [46,47]. Studies of interspecies association can prove effective in tracking ecosystem change as in the case of naturalised species that have been reported to generate positive influences, both natural-ecological and socio-cultural, and some of these benefits have been quantified [48].

However, in case of invasives, studies like ours can also provide opportunities for interventions before the establishment of permanent feedback loops. For example, at the time of data collection, another species with high invasive potential, *Leucaena leucocephala* was found to have a limited but significant impact; it was identified as the dominant invasive in at least two cliques (see Table 1), while loosely forming associations with *Prosopis juliflora* in other communities as a non-hub species (see Figure 7). Although it is not possible to say whether the invasive–invasive cliques it forms with PJ are more recent, or whether it has evolved into a more aggressive invader by forming its own dominant invasive cliques, we hypothesise that in few years it will break the present association, reduce the number of species, and may create a new clade of its own. The strength of the overall associations emerging from Table 1 and Figure 6 in Section 2.2 reflect mutual exclusion, as a form of association, among the invasive species, whereas Figure 7 (Section 2.3) reflects the extent of shared associations between invasive cliques. It should be recalled that the data presented in Section 2.2 only reflects individual locations whereas Section 2.3 combines all cliques across all locations, and therefore it brings out generic patterns that were not discernible in individual study sites. Competitive exclusion, particularly between invasive species is well reported in the literature where one invasive is shown to suppress another invasive species [49,50,51]. Some studies also indicate invasive meltdowns where a swarm of invasives can completely overtake native communities over a period of time [52]. However, in this study, we observed that while PJ and LC form exclusive cliques in individual study sites (Section 2.2), in the overall combined data presented in Section 2.3 we observed that there is a cohort of species that form transient associations shared by these two strong invaders (yellow-shaded region in Figure 7) and invasive exclusion is moderated by the presence of these transient associations. However, in terms of network topological properties such as module size and edge weights, it is quite evident that the invasive-specific associations (blue-shaded region in Figure 7) are stronger than the shared transient (yellow) associations. This observation could inform conservation planning as the existence of invasive-specific associations indicates that (a) some species clusters or locations are vulnerable and thus more likely to be colonised by invasives, and (b) once such associations have formed, it could also be harder to eradicate the invasives and restore native vegetation.

To understand or predict urban forest fragment communities and identify key species of concern, it is first essential to identify major groups of species that exist together or exclude each other as has been done in this study. However, the present work is cross-sectional, a single slice in time. Furthermore, the present investigation is focused on compositional patterns identifying cliques and associations between species, and we hope to pave the way for investigations into causal elements that lead to the formation of these communities. Therefore, there is a need to extend this study to understand the underlying processes that drive the formation of these assemblages—particularly plant functional traits that significantly affect the outcomes of competitive trade-offs between native and invasive plants. Further work is also needed to delineate the relative importance of landscape features at fine scales and competitive interactions in driving these assemblages in the urban context. This forms part of our ongoing work on Delhi Ridge and we are in the process of investigating the role played by landscape heterogeneity in explaining the associations.

In summary, we present a novel approach that uses complex network analyses to detect communities and explore associations between different species of plants more specifically to identify clusters formed by invasive species. From a management perspective, although we do find evidence for interventions to contain emergent invasives such as *Leucaena leucocephala* before they become more established in the ecosystems, a more extensive temporal analysis on similar lines needs to be performed in order to identify patterns that may assist in containing the spread of established invasives such as *Lantana camara* and *Prosopis juliflora*. This study is part of a long-term vegetation monitoring programme, and the early results on vegetation associations have been presented here. Further work on the interaction between different biotic and abiotic filters would provide insights into community assembly in the urban context.

## 4. Material and Methods

### 4.1. Site Selection

The South Delhi Ridge consists of multiple forest fragments that represent residual woodlands of Aravalli vegetation in the urban cosmopolitan city of New Delhi, namely, the National Capitol Territory (NCT), Delhi, India. For this study, all forest fragments >1 sqkm were included, leading to the identification of six study sites, as depicted in Figure 1. The first panel (Figure 1A) shows the map of New Delhi indicating each of these six study areas, namely, Hauz Khas (HK), Jahanpanah City Forest (JCF), Mehrauli Forest (MED), Tughlaqabad Forest (TUQ), Sanjay Van (SV), and Jawaharlal Nehru University (JNU). The climatic conditions of all six study areas are similar with rocky terrain (Pahari zone/Denudational Hills) and same soil type of Aravalli Quartzitic origin [53]. All forest fragments have the same nationally classified Forest Type, i.e., Northern Tropical Thorn Forests.

### 4.2. Vegetation Sampling

The primary vegetation survey data for this study was collected from a standardised ecological survey of six forest fragments in the South Delhi region, using the plotless sampling technique, i.e., line transect [54,55]. Line transects of 250 metres each were placed systematically inside each of the six identified study sites with an a priori design using Google Earth Pro. The transects were placed with a minimum distance of 100 m from each other, and from boundary walls or jogging tracks to minimise pseudo-replication and edge effects, respectively, as shown in Figure 1B. The alignment of the transects was opportunistic to avoid unscalable obstacles and geographic features. Transects that were found to be in modified landscape features, such as gardens, lawns, nurseries, buildings and other similar sites, were removed. All remaining transects were treated as sampling units, these were geo-referenced and subsequently used as a sampling guide for the entire vegetation survey. The number of transects varied depending upon the size of the patch and the area with built-up structures in each location. The starting points and bearing for each transect were accessed using a handheld GPS and the transect length was measured with the help of a Pedometer with a 3D motion sensor with predefined stride length.

### 4.3. Species Identification

For each site, the name of the species, number of individuals, girth at breast height (GBH) and canopy cover was recorded. It is important to point out here that some of the species names have changed during and after this study was conducted but to avoid confusion across all the published literature, nomenclature as per *Flora of Delhi* (1963) was followed [56]. Native and introduced species were categorised by referring to this key floral text. The categorisation of invasive species was done based on a list of alien invasive species of India published by the National Biodiversity Authority of India, under the Ministry of Environment Forests and Climate Change, Government of India [57]. Reference collections were made and herbarium samples were deposited in the Ecology Laboratory at the Ambedkar University Delhi for follow-up in case of difficulties with identification. In all, the data include presence/absence and abundance information from 57 woodland plant species, based on their representation in a total of 94 transects of 250 metres each.

### 4.4. Multivariate Data Analyses

Species richness was measured across the six sampling sites using rarefaction techniques followed by construction of distance dendrograms for each site, using the *vegan* package (version 2.5–7) in R-studio (Version 1.3.959). The Bray Curtis dissimilarity index along with a linkage method, the unweighted pair group method (UPGMA) was used for performing cluster analyses [49]. Species association and exclusion patterns were tested by Pearson correlation test (alpha = 0.05) between the relative abundance of both invasive species using *ggpubr* library in R-studio (Version 1.3.959) [58]

### 4.5. Network Analyses

Species presence–absence matrices were converted to structured information file (SIF) files using our in-house webserver NEXCADE [59]. Thus, each study area network dataset was comprised of an unweighted edge list. An edge list is a data structure used to represent a graph as a list of its edges. Unweighted edge lists are therefore two column matrices that directly connect nodes for each edge. In this case, each row in the edge list represented the physical location (or transect ID) and the name of the identified species, respectively. The SIF edge lists were exported for visualisation in Cytoscape version 3.9 [60]. Community detection was performed by parsing each study area network through algorithms that identify densely connected regions using topological parameters. For this, we used the MCODE and GLay network partitioning algorithms, which enable versatile community structure identification as well as graph layout functions for network clustering and structured visualisation [61,62]. For each of the six forest community networks, this led to the identification of node clusters, which we called ‘cliques’. The species with the highest degree in each clique were treated as ‘hubs’ or key species of each clique and all study area networks were compared in terms of clique composition. Finally, all the cliques comprising the two major invasive species (*Lantana camara* and *Prosopis juliflora*) were superimposed in order to explore and assess the extent of the fidelity of species associations formed by these invasives. This was done using the Markov cluster algorithm or MCL, an unsupervised cluster algorithm for networks based on the simulation of (stochastic) flow in graphs [63].

## 5. Conclusions

There has been considerable interest in the last two decades in understanding the ecology of urban ecosystems that were previously understood to be degraded as they were poor representations of the original vegetation. While these urban ecosystems are dominated by non-native and invasive species, there is renewed interest in these modified or ‘novel ecosystems’ as they continue to provide ecosystem services and value to the city. Urban woodlands and forest remnants in Delhi consist of a combination of species that range from species native to the Aravalli, to those that were introduced to Delhi purposefully during the urbanisation, to species that have escaped from cultivation. Of all these groups, invasive species are seen to be a significant challenge for conservation efforts because they are known to displace natives or cause declines in native species populations. Using a network approach in this study, we identified distinct communities of plants among the vegetation survey data from six urban forest fragments in the South Delhi Ridge of the Aravalli region. Species that were found to occur together significantly more often than by random chance, represent co-occurrence patterns. In contrast, species that were not found to grow together on the same-line transects reflected a tendency to consistently avoid each other, thereby suggesting a pattern of exclusion.

Importantly, we identified co-occurrence as well as exclusion patterns among species across the six urban forest communities. Despite overlaps, these communities constitute invasive–invasive, invasive–native and native–native associations in each of the woodland patches investigated. We found that the invasive species *Lantana camara* and *Prosopis juliflora* form species assemblages or ‘cliques’ that are mutually exclusive across all sites, in the sense that these two major invaders do not form associations with each other. As such, these two major invasive species form invasive–native and invasive–invasive associations that seem consistent across different forests, indicating the formation of new stable associations in Delhi’s woodlands, and supporting the widely held notion of novel ecosystems in urban ecology. This work indicates that community identification algorithms can find applications in pattern analysis in vegetation ecology and may provide an altogether new way of investigating species associations using networks. In summary, our findings have implications in the conservation of these urban forests and this work highlights a new application of network approach for identifying species associations and visualisations that could be explored further for its efficacy in vegetation analysis.

## Figures and Tables

**Table 1:** Distinct communities within urban forests show distinct ‘native–invasive’ and ‘native–native’ cliques. Invasive species are marked in bold letters.

Site	# Species	Clique	(Hub) Species in Each Community/Clique
**SV**	42	1	***Leucaena leucocephala***, *Cassia fistula*, *Pongamia pinnata*, *Ailanthus excelsa*, *Carissa spinarum*
		2	*Azadirachta indica*, ***Lantana camara***, *Grewia tenax*, *Holoptelea integrifolia*, *Dalbergia sissoo*
		3	*Adhatoda vasica*, *Capparis sepiaria*, *Balanites roxiburgii*
		4	*Capparis decidua*, ***Prosopis juliflora***, *Diospyros cordifolia*, *Maytenus senegalensis*
		5	*Tectona grandis*, *Acacia senegal*, *Ziziphus mauritiana*, *Prosopis cineria*
**TUQ**	31	1	*Balanites roxiburgii*, ***Prosopis juliflora***, *Capparis sepiaria*
		2	*Acacia nilotica*, *Cassia fistula*, *Acacia lecucophloea*, *Prosopis cineraria*
		3	*Grewia tenax*, *Holoptelea integrifolia*, *Pongamia pinnata*, *Ziziphus nummularia*, *Diospyros cordifolia*
		4	*Azadirachta indica*, *Capparis decidua*, *Adhatoda vasica*
			***Lantana camara***, *Bougainvillea spectabilis*
**HK**	50	1	*Senna siamea*, *Pongamia pinnata*, *Capparis sepiaria*, *Ehretia laevis*
		2	***Leucaena leucocephala***, *Morus alba*, *Adhatoda vasica*, *Acacia nilotica*, *Dalbergia sissoo*
		3	***Lantana camara***, *Diospyros cordifolia*, *Ziziphus nummularia*, *Azadirachta indica*
		4	*Prosopis cineraria*, *Cassia fistula*, *Grewia tenax*, *Capparis decidua*, *Murraya koenigii*, *Drypetes roxburghii*
		5	***Prosopis juliflora***, *Bombax malabarica*, *Milletia peguensis*, *Ailanthus excelsa*, *Terminalia arjuna*
**JCF**	49	1	*Prosopis juliflora*, *Capparis sepiaria*, *Carissa spinarum*, *Azadirachta indica*
		2	*Holoptelea integrifolia*, *Pongamia pinnata*, *Albizia amara*, *Cassia fistula*, *Senna siamea*, *Tectona grandis*, *Prosopis cineraria*
		3	***Lantana camara***, *Ziziphus nummularia*, *Bombax malabarica*
**MEH**	37	1	***Lantana camara***, *Adhatoda vasica*, *Acacia nilotica*, *Holoptelea integrifolia*, *Pongamia pinnata*, *Cassia fistula*
		2	*Cassia tora*, *Ziziphus nimmularia*, *Acacia lecucophloea*, *Grewia tenax*
		3	*Abutilon*, *Capparis sepiaria*, *Capparis decidua*
		4	*Azadirachta indica*, ***Prosopis juliflora***
**JNU**	42	1	*Balanites roxiburgii*, ***Lantana camara***, *Ziziphus nummularia*, *Ziziphus mauritiana*
		2	*Capparis decidua*, *Adhatoda vasica*, *Azadirachta indica*, *Acacia lecucophloea*
		3	***Prosopis juliflora***, *Capparis sepiaria*, *Diospyros cordifolia*

## Data Availability

The data is available from the authors upon request.

## Supplementary Materials

The following supporting information can be downloaded at: https://www.mdpi.com/article/10.3390/plants11040541/s1, Table S1: Species Name Codes.
